# Schizophrenic or blind but not both

**DOI:** 10.1192/j.eurpsy.2022.2049

**Published:** 2022-09-01

**Authors:** F.Z. Chamsi, I. Katir, A. Korchi, S. Belbachir, A. Ouanass

**Affiliations:** 1 Psychiatric hospital Ar-razi, Psychiatry, Salé, Morocco; 2 Ar-razi University Psychiatric Hospital of Salé, Salé, Morocco, Psychiatry, Salé, Morocco; 3 Hôpital psychiatrique ARRAZI de Salé, Psychiatrie, Salé, Morocco; 4 hopital arrazi de sale, Psychiatire, sale, Morocco

**Keywords:** blindness, congenital, schizophrénia

## Abstract

**Introduction:**

Although visual impairment appears to be a risk factor for schizophrenia, early blindness may be protective. It’s a phenomenon that has puzzled even the smartest scientific brains for decades. It might surprise you: no person born blind has ever been diagnosed with schizophrenia.

**Objectives:**

The aim of this research is to discover the relationship between schizophrenia and congenital blindness and whether there is a protective gene and whether visual perception is an essential stage in the onset of diseases itself.

**Methods:**

It’s a case study of a family consisting of 13 brothers and sisters, three of whom were blind at birth, three with schizophrenia. We proceeded with a study of the medical files of all the schizophrenic patients and also ophthalmological exams for all the family members.

**Results:**

Preliminary observational analysis of this clinical case suggests the following hypothesis: the presumed protective role of congenital blindness against schizophrenia. Moreover, the ophthalmological exams showed no visual impairment in schizophrenic patients. The bibliographic research has objectified more than three recent studies in this direction.

**Conclusions:**

The relationship between schizophrenia and congenital blindness is still unrecognized and controversial. Several studies are done in this neurodevelopmental field but so far there has been no assertion nor confirmation of the suggested hypothesis. More research is needed.

**Disclosure:**

No significant relationships.

